# Measurement of Work-Life Balance: A Scoping Review with a Focus on the Health Sector

**DOI:** 10.1155/2023/3666224

**Published:** 2023-03-04

**Authors:** Mohamad Alameddine, Nabeel Al-Yateem, Karen Bou-Karroum, Heba Hijazi, Alounoud Al Marzouqi, Samir Al-Adawi

**Affiliations:** ^1^College of Health Sciences, University of Sharjah, Sharjah, UAE; ^2^Health and Workforce Studies Research Group, Research Institute of Medical & Health Sciences Sharjah, University of Sharjah, Sharjah, UAE; ^3^Mohammed Bin Rashid University of Medicine and Health Sciences, College of Medicine, Dubai, UAE; ^4^Department of Health Management and Policy, Faculty of Health Sciences, American University of Beirut, Beirut, Lebanon; ^5^Department of Health Management and Policy, Faculty of Medicine, Jordan University of Science and Technology, Irbid, Jordan; ^6^Department of Behavioral Medicine, College of Medicine and Health Sciences, Sultan Qaboos University, Muscat, Oman

## Abstract

**Background:**

There is an agreement on the importance of measuring work-life balance, especially after the COVID-19 pandemic. However, the available tools to do so are not sufficient to address all dimensions, contexts, and professions.

**Aim:**

The article reviews existing instruments that have been widely utilised to tap into the breadth and depth of work-life balance. *Evaluation*. This is a perspective scoping review guided by PRISMA-ScR guidelines. Articles reporting on the measurement of work-life balance were reviewed. The authors performed the review based on agreed-upon search terms, inclusion and exclusion criteria, search databases, and the data extraction process. *Key Issues*. The existing tools appear to have divergent underpinning theoretical models, factors, structural/psychometric properties, and the number of accumulated citations. The existing tools also varied in terms of their target sector, with limited tools available for the analysis of work-life balance among healthcare professionals. We argue that while the existing tools provide a general base for the work-life balance measurement, it would be imperative to adjust those tools to the specific cultural and professional contexts. Future work-life balance measures should consider the changes imposed by atypical or disruptive events that have the potential to alter work-life balance, such as in the case of the COVID-19 pandemic. The onus is on researchers and policymakers to work collaboratively in each context to adapt, implement, and evaluate those tools as they become integrated into the matrix of labour market assessments in the future.

**Conclusions:**

The article highlighted current gaps and improvement opportunities in the work-life balance measurement field. *Implications for Healthcare and Nursing Management*. The maintenance of work-life balance will remain an issue for years to come. Ensuring comprehensive and context-specific measurements would be essential to guide the evidence-based recommendations necessary to support the workforce across the various sectors of the economy in the future.

## 1. Introduction

The global pandemic (COVID-19) is likely to act as a catalyst for changes as it has occurred in previous pandemics [1]. For example, there is a suggestion that the “black death pandemic” that peaked between 1347 and 1351 acted as a strong precursor to the “reawakening” or renaissance of societies in Western Europe, a feat that subsequently spread technological and social changes throughout the world [2]. Besides the spread of digital and online technologies, one of the many outcomes of the COVID-19 pandemic is the impact on work-life balance. As a part of the social distancing measures to address COVID-19, populations around the world are increasingly working from home [3]. The world's swift transition to remote work has obliged a sector of the population to work in an unplanned context and in many cases, without prior experience [4]. A recent study revealed that 22% of individuals living with young children had difficulties concentrating on their jobs all or most of the time [4]. It is evident that working from home increases work intensity as well as work-life conflict, affecting workers' overall well-being [4].

The interest in assessing and maintaining work-life balance has strong historical underpinnings. The first industrial revolution, which took place in Western Europe and North America between 1820 and 1840, partially eroded the traditional mode of living and ethos of life and gradually demarcated the division of labour, gender role, and what constitutes work and nonwork [5]. The cultural and economic lifestyle that was used to sustain the preindustrialised period was replaced by the growth of urbanity and an economy that strongly hinges on manufacturing processes [5]. The work in the manufacturing sections was for a designated time of the day or season. Within such a context, the division between work and nonwork was solidly consolidated. More recently, with the advent of information technology and “Internet culture,” work-life balance is increasingly blurred since work could be easily performed in a domestic setting [6, 7]. This paradigm shift calls for a critical appraisal of the current work-life balance research in terms of concepts and measures.

Globally, there has been an increasing interest among researchers and academics in assessing work-life balance [8]. This growing interest is driven by the significant role of work-life balance in overall life satisfaction, which is in turn associated with improved health and professional performance outcomes [9, 10]. Better work-life balance improves organisational productivity and commitment, enhances job satisfaction, and reduces absenteeism and turnover intentions [11]. The published literature has identified several antecedents of work-life balance including but not limited to reduced work support, work schedule, work overload, and role conflicts [12]. The main work-family conceptualisations focus on balance, enrichment, and conflict. Work-family balance is defined as the lack of conflict or interference between the work and family roles [13]. Work-family enrichment is a positive way whereby work and family interact resulting in enrichment between the two domains [14]. Work-family conflict is thus an interrole conflict whereby the pressure from the work and family domains are mutually incompatible [15].

The healthcare industry is one of the most dynamic sectors worldwide. The demographic and epidemiologic transitions are the main driving factors for the growing demands for healthcare services [16]. To meet the increased demands, a skilled and well-trained workforce is essential. Such developments were associated with high workload, long working hours, shortage of staff, and lack of flexibility. This demanding environment has led to an imbalance between work and family demands, resulting in undesirable outcomes at the individual, family, and organisation levels [17]. A recent study from Ireland revealed that 73% of doctors were feeling the strain of work-life imbalance [18]. Crucially, the COVID-19 pandemic has heightened work-life imbalance among healthcare professionals [19]. Given that healthcare professionals are at the forefront of the global fight against the virus, they often do not have time to seek support from family and friends to relief stress and reinforce resilience [19]. As such, international concerns were raised regarding the well-being of healthcare professionals, calling for interventions that enhance the balance between work and nonwork demands [20].

There is no single definition or a unified consensus to measure for work-life balance in the literature [10]. Instead, several definitions and measures are available, including defining work-life balance as multiple roles, equity across multiple roles, satisfaction between multiple roles, the fulfilment of role salience between multiple roles, a relationship between conflict and facilitation, and perceived control between multiple roles [10]. These different conceptualisations have varying degrees of success within the literature [10]. Having multiple definitions affects measurements and, consequently, the corrective measures taken. Furthermore, it will not allow comparing work environment situations in different contexts and countries and thus scatter the efforts to suggest the evidence-based policy and practice improvements that would improve the work environments at the global level.

## 2. Scoping Review Aim

The present narrative aims to review existing instruments that have been widely utilised to tap into the breadth and depth of work-life balance. Specifically, this present quest is to analyse the tools across various dimensions and critique their strength and limitations, hoping that this will pave the way for more focused discussions and serious efforts to solidify one comprehensive definition and, thus, better measurement tools and effective interventions.

## 3. Methodology

This is a perspective review guided by a quick narrative review. Articles reporting on the measurement of work-life balance were reviewed, considering the psychometric properties of the scales and their heuristic values.

### 3.1. Eligibility Criteria and Selection of Sources of Evidence

The inclusion criteria were as follows: articles published in English, articles focusing on work-life balance dimensions and constructs, and articles revealing the development and validation of work-life balance measurements. All available years were searched. Finally, the search included studies investigating work-life balance within the healthcare professional's population. Articles were excluded if they were not in English language.

### 3.2. Information Sources and Search Process

An initial search was carried out using the keywords: “work-life balance,” “work-family conflict,” “work-life interference,” “work-family balance,” “work-family interference,” “work-nonwork balance,” and “work-life enhancement.” The search was carried out in PubMed, EMBASE, and PsycINFO and covered articles published up to October 2020. Reference lists and keywords of reviewed articles were used to identify additional relevant articles and extend the initial search of the literature.

### 3.3. Data Charting and Summarizing

**Figure 1:**
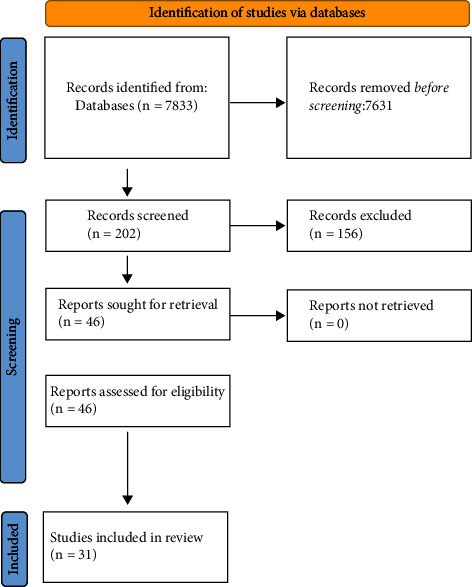
Databases search and the article reduction flowchart.

The title and abstracts were first assessed by two authors to identify relevant articles. The authors met regularly online to resolve any disagreements about whether articles fulfilled the inclusion criteria. However, in all cases, a consensus was reached. Data extracted from each eligible article included the author(s), the year of publication, the number of items, the target sector, psychometric properties, the country of development/validation, and a brief description of the measure and the main constructs within it. We further categorised each work-life balance measure into one of the eight work-life balance dimensions. The Scopus database was used to determine the number of citations for each of the selected articles as an indicator of the scale popularity and use throughout the literature.  Figure 1 illustrates the PRISMA databases search and the article reduction flowchart.

## 4. Results

Database searches yielded 202 articles related to work-life balance measurements. After the title and abstract review, 46 articles were selected and obtained. Further scrutiny was conducted by the authors according to the inclusion criteria. Our search uncovered the existence of 31 measurement tools dating back to 1983 with the most recent one published in 2020.  Figure 1 illustrates the database search results and the article reduction process.

The review revealed that during the early decades of work-nonwork balance research, measures focused on work-family balance as a unidimensional construct [21]. This first generation of scales did not distinguish between the directions of conflict [22]. Later, work-life balance scales evolved from the initial focus on the single direction to two directions, which are work interference with nonwork activities and nonwork activities interference with work. This supported the understanding of the antecedents and consequences of the two different conflict forms [22].

It is important to note that the work-family theme was overemphasised in the literature, which resulted in the confinement of the work-life balance research. Work-life balance measures focused only on the work-family conflict, suggesting that “life” means “family.” However, life is segmented into several domains other than family life, including leisure life, social life, community life, and financial life [9]. By the end of the 1990s, researchers argued that the work-family balance is only a subtheme under the umbrella of the work-life balance construct and started exploring new nonwork areas beyond the family [23]. Additionally, this shift in terminology aims at including employees who are not parents but are engaged in different nonwork activities. Subsequent scales measured different constructs, broader than work-family balance. This generation of scales includes the domain-level measurement, providing diagnostic information on which life domains are affected by work demands [23]. For example, work might interfere with only one aspect of life such as health or education, but not with other aspects such as leisure and community involvement.

Other scales assessed the work-family conflict from a bidirectional perspective with three different forms of interference: (1) time-based interference which refers to the time devoted to one role, making it difficult to meet demands in other roles [24–26]; (2) strain-based interference refers to the tension and fatigue created by one role, making it difficult to participate in another role [27]; and (3) behaviour-based interference refers to when behaviours required in one role are incompatible with behavioural expectations in another role [28]. A study by Clark, Early, Baltes, and Krenn differentiated between behaviour-based conflict and behaviour role conflict, defining the latter as “the specific instances when work interferes with family or family interferes with work (irrespective of whether the same or different behaviours are expected in each domain)” [27]. However, behaviour-based conflict only occurs when different behaviours are expected in the different roles and the person fails to adjust his/her behaviour to comply with the different expectations of each role [27]. This work-family behavioural role conflict scale provides a better understanding of how individuals perceive their work-family conflict episodes.

One scale by Carlson and Frone has focused on the internal-external dimension of work interference with family and family interference with work [28]. This scale has differentiated between the internal (psychological) and external (behavioural) dimensions of work-family interference. The internal interference represents internally generated psychological preoccupation with one domain in life while within the boundaries of another domain. The external interference represents externally generated demands in one role which prevents participation in another role.

More recently, researchers included the positive side of the work-life interface and the advantages of achieving a balance between the two, represented in terms such as work-family enhancement, spillover, enrichment, and facilitation [29, 30]. This positive side represents the extent to which experiences in one life domain improve the quality of another domain. Subsequently, work-life enrichment has been differentiated from spillover. Wayne defined spillover as the gains (e.g., values, behaviours, and skills) acquired in one domain and used in the other domain, but do not necessarily enhance the performance in the other domain [31]; whereas, enrichment occurs when the gains from one domain enhance functioning in other domains [21]. Another study suggested measuring work-family balance by using work-family conflict and work-family enrichment scales [32]. This “components approach” captures a variety of types of work-life conflict and enrichment to ensure adequate coverage of all experiences that may contribute to work-life balance. This allows greater clarity and accuracy in describing antecedents of work-family balance, as it is highly unlikely that work-life conflict have identical antecedents as work-life enrichment. Similarly, since it is highly plausible that work-life conflict and work-life enrichment have different consequences, this approach helps in understanding salient outcomes such as workers health [32].

Moreover, scholars differentiated two constructs of work-life balance, the psychological construct which is held in the mind of a focal person or the rational construct which can be observed by others. For example, Grzywacz and Carlson used the rational construct and defined work-life balance as “the accomplishment of role-related expectations that are negotiated and shared between an individual and his/her role-related partners in the work and family domain” [32], whereas Valcour used the psychological construct and defined work-life balance as “an overall level of contentment resulting from an assessment of one's degree of success at meeting work and family role demands” [33].

It seems difficult to measure work-life balance due to the lack of consistency in construct definitions. The diverse conceptualisations of work-life balance ranging from satisfaction with work and family domains to positive spillover have all been labeled “work-family balance” and used interchangeably in the literature. Another concern about work-life balance is that almost one-fifth of the measures are single items that cannot capture complex constructs [34].  Table 1 presents an overview of measurement scales used to survey work-life balance.

### 4.1. Work-Life Balance among Healthcare Professionals

**Figure 2:**
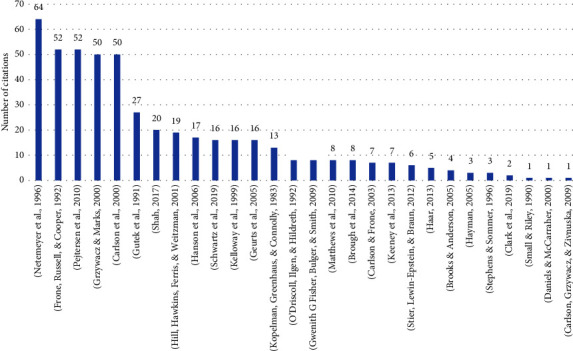
Graph showing number of citations per year for work-life balance instruments. ^*∗*^Studies that included healthcare professionals in their sample upon development and validation of the scale.

The most cited scales in studies measuring work-life balance among healthcare professionals are the Work-Family Conflict Scale which constitutes of 10 items measuring the bidirectional perspective of work-life balance [47], the Work-Life Climate Scale which constitutes of 8 items [35], and an 18-items scale measuring the time-, strain-, and behaviour-based work interference with family and family interference with work [24]. Other studies used scales such as the Copenhagen Psychosocial Questionnaire II—Work-Family Conflict Scale [45], the Work-Family Conflict Scale [50], the Work-Life Balance Scale [43], and the Work-Family Positive Spillover [56]. Furthermore, some studies included healthcare professionals in the sample upon the development and validation of the scales (highlighted in Figure 1). These include the Work-Family Conflict Scale [25], the Work-Life Checklist [40], the Quality of Nursing Work Life [38], the Work Interference with Life Domains Scale [23], the Work-Life Balance Measure [36], and the Work-Life Balance Scale [53].  Figure 2 shows number of citations per year for work-life balance instruments.

It was noted through our review that these work-life balance scales failed to represent all healthcare professional groups such as physicians and pharmacists, with few scales focusing on specific groups such as nurses and psychologists. One study showed that out of all healthcare professionals, physicians reported the poorest work-life balance behaviour followed by nurses and physician assistants [57]. Developing work-life balance scales with consideration of the various healthcare professional groups is therefore essential as frontline medical workers are different than behind the desk workers. The health sector is approaching a tipping point as occupational burnout and work-life conflicts are becoming more prevalent among healthcare professionals [35]. Particularly in times of pandemics and crisis, healthcare professionals are the most vulnerable [58]. As such, it is essential to develop more diagnostic and actionable measures for targeted interventions in the healthcare sector. Since work-life balance is closely linked to several clinical and organisational outcomes, including work-life balance surveys in the routine safety culture assessments is crucial [35].

## 5. Discussion and Future Agenda

The present narrative review suggests that research on the quantification of a work-life balance focusing on healthcare professionals is still nascent. As the traditional dichotomy between work and home is increasingly blurred, more research studies on these endeavours are therefore warranted to keep up with the marching time and evolving social revolution that owes its onset to the technological innovation and social changes entailed. The present narrative review has unearthed the trend in the literature that are described herein in tandem. These points should be seen as groundwork for further scrutiny.

First, there is heterogeneity in the definitions of work-life balance and the approach to quantification. Thus, when researchers measure work-life balance, they use different scales that assess different concepts and dimensions. For example, some scales measure work-life balance from a unidirectional perspective such as the “Work-Family Balance Scale [41].” In contrast, other researchers measured the bidirectional construct of work-life balance with a focus on the spillover effect of one to the other. The diverse definitions and conceptualisations of work-life balance are problematic as a precise definition is essential to support proper measurement. Also, the work-family interface has been greatly emphasised in the literature [59], whereas the various aspects of life beyond family have received scant empirical scrutiny. For example, the experiences of individuals without children were not well captured despite the fact that there is a significant growth globally on marrying or cohabitating late despite having a steady career [60]. Little emphasis was placed on linkages between work and other aspects of individuals' nonwork areas as the time one spent out of the realm of work such as community involvement, volunteering, and other aspects of daily routine such as self-care and leisure. As such, future scales should consider a broader conceptualisation that is more representative of employees' experiences at the intersection of work and a variety of life roles that constitute the nonwork domain.

Second, although there are multiple scales available to tap into work-life balance, their application has contextual differences. It was noted through our review that the literature on work-life emanates were mostly conducted among 20% of the global population residing in Western Europe, North America, and the pocket of countries in the Pacific Rim. However, there is a dearth of culturally sensitive scales for 80% of the global population where there is a spurt of industrialisation, acculturation, and urbanisation, and therefore, traditional modes of living are increasingly untenable [61]. The present narrative review suggests that only a few studies have been developed and validated on work-life in non-Western countries with a few exceptions [62]. The work-life balance scales developed and tested in the US might not be valid in other countries. Norms and cultural values play a significant role in shaping the work-life interface [63]. Societal or national culture would affect an individual's experiences in work and life domains. For example, scales examining the experiences of Indian employees emphasised the effect of gender on the work-life interface. Their arguments were based on a thorough assessment of the Indian culture where the idea of the male provider role is persistent, in contrast to the Western culture. Another important cultural dimension to consider is individualism/collectivism, describing the nature of relationships among people, that is, whether people focus on individual goals and believe they are independent (individualists) or whether they focus on group goals (collectivists). Humane orientation is also a cultural dimension describing the degree to which societies reward and encourage individuals for being friendly, caring, and generous to others. Cultures with a high degree of humane orientation values may provide better support for individuals in managing the work-life interface than members in cultures with a low degree of humane orientation values [62]. Given that the work-life interactions are entrenched in larger societal contexts including gender roles, cultural values, and national policies, the expansion of work-life balance scales is merited. Future scales need to account for the diverse array of cultures and increase understanding of cultural influences on the work-life balance. These culturally sensitive scales would in turn inform decision makers on the best work-life practices and policies that are culturally appropriate [62, 63].

Third, research on work-life balance supports the existence of a positive spillover effect, arguing that multiple roles can be beneficial [21]. Positive spillover has been significantly associated with improved emotional, mental, and overall health. Several scales have included both ways of spillover, the negative and the positive spillover [21]. However, research on the spillover effect of the work-life interface is less developed than research on work-life conflict. The spillover between work and life domains is inevitable. Therefore, all work-life balance measures should include items that capture the spillover construct of the work-life interface.

Fourth, it was noted that almost all work-life balance scales were tested among a sample of employees from various occupations. We hereby argue that future work-life balance scales should develop items specific for each work sector, taking into account the requirements of each occupation. Certain industries and occupations require long working hours in challenging circumstances such as roles in healthcare and law enforcement, making it difficult to balance work and life domains. It is also evident that healthcare professionals and frontline workers are the most susceptible ones during infectious disease outbreaks, emphasising the need for a specific work-life balance scale for the healthcare sector. Furthermore, this allows organisations to implement targeted interventions and policies to enhance the work-life balance of their employees. Therefore, it would be worthwhile to develop sector-specificwork-life balance scales.

Finally, the emergence of coronavirus disease 2019 (COVID-19) pandemic has resulted in significant changes in peoples' work and personal lives, as well as their roles within their families. During the pandemic, critical measures for COVID-19 prevention and control have been implemented including lockdowns, social distancing, and closing of schools and public institutions [64]. As such, adults and children have been forced to stay at home for an unknown time. As the home became the new workplace, as well as the new school, it is more challenging for families to successfully coordinate work and family obligations. The COVID-19 pandemic had brought back the gendered division of labour whereby women bore the burdens of the household chores, children, and emotional labour. This was evident in Iceland, a country on the top of the Gender Gap Index, where mothers expressed their frustration with the uneven division of labour during the pandemic [65]. In these difficult times and when work-life balance research is surging, an important question arises: how can we shape work-life balance scales to cover the new changes imposed by the pandemic? How can we identify the gendered interactions of work and life domains?

The present narrative review on work-life balance and specifically on its quantification provides some insights from what appears to be nascent research work-life balance and highlights important gaps in research studies that should lay the groundwork for further scrutiny. Specifically, we discussed the heterogeneity of work-life balance definitions and constructs. Eight dimensions of work-life balance have been identified: unidirectional perspective; bidirectional perspective; time-, strain-, and behaviour-basedwork-life conflict; work-life enrichment/enhancement; work-life spillover; internal vs. external work-life balance; domain-level measurement; and bidirectional perspective with a focus on behavioural role conflict. Reducing much of the heterogeneity in work-life balance definitions has important practical consequences for how work-life balance is to be measured and for how scales are to be designed. In addition, it is essential to develop contextualised and sector-specificwork-life balance scales while enhancing international knowledge sharing and collaboration.

### 5.1. Implications for Healthcare and Nursing Managers

As the conventional barrier between work and home becomes increasingly blurred, healthcare managers and leaders, including nurse leaders, should focus on quantifying and measuring the work-life balance. However, as there is a heterogeneity of work-life balance definitions and quantification methods, managers and leaders in the healthcare sector should tailor the measures to the healthcare system's circumstances and goals. Work-life balance scales should also be constructed to account for unexpected situations such as pandemics and public health emergencies. It should also take different genders and their individual needs into account.

Furthermore, the majority of work-life balance scales were general and targeted employees from a variety of occupations. It is recommended that healthcare leaders develop specific scales containing healthcare-specific items, considering the needs of healthcare professionals. The contextualisation of work-life balance measures is an important exercise that needs to involve nurses, nursing orders, and syndicates in a global discourse. Therefore, it is recommended that the International Council of Nurses endorse this as a priority area in the coming years.

Lastly and perhaps most importantly, listening to the voice of nurses in regard to their work-life balance precipitates a mandate to introduce evidence-informed improvements that would help nurses regain their balance while exercising their full potential. Therefore, it is pivotal that institutional leaders endorse an improvement system that is supported operationally to bridge the voice of nurses to actual changes in their work environments.

## Figures and Tables

**Table 1:** Overview of measurement scales used to survey work-life balance.

Themes	Names	References	Sample characteristics	Countries
Unidimensional construct of work-life balance	Work-Life Climate Scale	[35]	Healthcare workers	—
	Work-Life Balance Measure	[36]	Workers from industries such as public service, health, education, finance, manufacturing, and nongovernment organizations	Australia and New Zealand
	Work-Family Balance Measure	[37]	Full-time employees	—
	Quality of Nursing Work Life (QNWL)	[38]	Nurses	—
	Work-Life Balance	[39]	Employees from various job categories such as programmers, engineers, services, sales, consultants, project managers, and exempt professionals	United States
	Work-Life Checklist	[40]	Employees from finance, health, and social-service sectors	Britain
	Work-Family Conflict Scale	[41]	Employed students and technical professionals	—

Bidirectional (work interference with family and family interference with work)	Work-Life Balance	[11]	Employees of banking and information technology sector	India
	The Work and Family Conflict Scale (WAFCS)	[42]	Working parents	Australia
	Work-Life Balance	[43]	IT professionals	India
	Work-Family Conflict	[44]	Employees from different occupations such as blue-collar, while-collar, self-employed, employed in public sector, or has job authority	27 countries participated in the survey
	Copenhagen Psychosocial Questionnaire II—Work-Family Conflict Scale	[45]	Applied to various occupations including machinists, cleaners, clerks, mechanics, medical doctors and dentists, physical and occupational therapists, supervisors, media employees, academics, and social education workers	—
	Survey Work-Home Interaction NijmeGen (SWING)	[46]	Workers from a manufacturing company, postal office, a financial consultancy firm, primary schools, and a governmental institute in the service sector	European countries
	Work-Family Conflict Scale	[47]	Sample 1, elementary and high-school teachers and administrators	United States
			Sample 2, small business owners	
			Sample 3, real estate salespeople	
	Work-Family Interface	[48]	Full-time workers	United States
	Interrole Conflict Scale	[49]	Employees from a broad range of occupational categories	United States
	Work-Family Conflict	[50]	Psychologists	—

Bidirectional perspective with separate items for time, strain, and behaviour-based conflict	Work-Family Conflict	[26]	Employees in industries	—
	Work-Family Conflict Scale	[24]	Employees in a division of a state government agency	United States
	Work-Family Conflict	[25]	Employees from healthcare and a retail grocery organization, mainly nurses and in-store personnel	—
	Work-Family Conflict Scale	[51]	Employees at a large rehabilitation hospital (management staff and professional rehabilitation staff excluding consulting physicians), at a government agency, and at a scientific testing firm	—

Bidirectional perspective with a focus on behavioural role conflict	Work-Family Behavioral Role Conflict Scale	[27]	Participants held a wide range of jobs, primarily in management occupations (15%), office and administrative occupations (10%), sales (9%), and healthcare (9%)	United States

Domain-level measurement of work-life balance	Work Interference with Life Domains Scale	[23]	Participants represented diverse occupations including management, education, training, library, business and financial operations, healthcare practitioners (8%), arts, design, entertainment, sports and media, and architectural and engineering	—
	Work Spillover into Family Life	[52]	Bank executives	—

Bidirectional perspective with items for work-family enrichment/enhancement	Work-Life Balance Measure	[53]	Study sample included different professions such as architects, doctors, lawyers, lecturers, and scientists	India
	Work-Life Balance	[54]	Employees from a wide range of firms	New Zealand
	Work/Nonwork Interference and Enhancement Scale	[29]	Managers employed in a variety of organizations, departments, and industries	United States
	Work-Life Balance Instrument	[55]	Administrative and professional employees from a large university	Australia

Bidirectional perspective with items for work-family spillover	Work-Family Interface	[30]	Employees from several occupations	United States
	Work-Family Positive Spillover	[56]	Employees working for a large distribution center	United States

Bidirectional perspective with a focus on the internal-external dimension	Work-Family Interference	[28]	Full-time employees, with 53% held white-collar jobs	USA - New York

## Data Availability

The datasets generated during and/or analysed during the current study are available from the corresponding author upon request.
